# Innovations in Diabetes Care for a Better “New Normal” Beyond COVID-19

**DOI:** 10.1210/clinem/dgaa704

**Published:** 2020-10-12

**Authors:** Shivani Agarwal, Michelle L Griffith, Elizabeth J Murphy, Carol Greenlee, Jeffrey Boord, Robert A Gabbay

**Affiliations:** 1 Fleischer Institute for Diabetes and Metabolism, NY-Regional Center for Diabetes Translational Research, Albert Einstein College of Medicine, Bronx, New York; 2 Division of Diabetes, Endocrinology, and Metabolism, Vanderbilt University Medical Center, Nashville, Tennessee; 3 Division of Endocrinology, Zuckerberg San Francisco General: University of California, San Francisco, California; 4 Western Slope Endocrinology, Grand Junction, Colorado; 5 Parkview Health System, Fort Wayne, Indiana; 6 Joslin Diabetes Center, Harvard Medical School, Boston, Massachusetts

**Keywords:** healthcare delivery, diabetes care, innovation, alternative models of care, COVID-19, healthcare delivery, diabetes care, innovation, alternative models of care, COVID-19

## Abstract

The coronavirus disease pandemic has created opportunities for innovation in diabetes care that were not possible before. From the lens of this “new normal” state, we have an opportunity to rapidly implement, test, and iterate models of diabetes care to achieve the quadruple aim of improving medical outcomes, patient experience, provider satisfaction, and reducing costs. In this perspective, we discuss several innovative diabetes models of care which promote collaborative care models and improve access to high-quality specialty diabetes care. We discuss ongoing threats to diabetes care innovation, and offer practical solutions to foster evolution and sustain current strides made during the pandemic.

## A Force of Change

The “new normal” has been coined as a term to denote the major shift in paradigm that coronavirus disease 2019 (COVID-19) has forced around the globe. In health care, the “new normal” has fostered innovation and allowed for real-world testing of care models in unprecedented ways. In this watershed moment for health care systems around the world, real change may be possible to create a better new normal beyond the pandemic if careful steps are taken to promote advocacy and ensure sustainability.

For diabetes, which affects half a billion people worldwide, the thrust toward innovation has long been overdue. Achieving optimal glycemic control on a large scale has major implications for population and economic health, yet less than 50% of people with diabetes meet glycemic targets (1, 2). Before the pandemic, innovative models of diabetes care were slowly gaining recognition, aiming to achieve improvements in medical outcomes, patient experience, health care provider satisfaction, and reduction in costs. However, typical roadblocks existed to impede meaningful change. With COVID-19, implementing alternative models of care on swift and iterative cycles became the norm.

In 2018, the Endocrine Society convened a taskforce to examine and promote innovative models of care in diabetes. In this perspective, the members of the taskforce review several models of care, with which they have experience and which have potential to become standards of care in the new normal. We discuss the current challenges to widespread adoption of new models of care. Last, we provide potential solutions to foster innovation and create pathways for sustainability.

## Innovations in Diabetes Care

We review in detail here models that each of the Taskforce members have had success with. Specifically, these models focus on improving access to high-quality specialty endocrinology care through care coordination and opening lines of communication.

Clinically, each of these models is applicable to both type 1 and type 2 diabetes. Although historically, some of these innovations have been more popular to a particular type of diabetes, they are becoming increasingly relevant across the spectrum because there is wider distribution of both types of diabetes across the lifespan and technological treatments traditionally reserved for type 1 diabetes are now being used for type 2 diabetes as well.

### Telemedicine

Telemedicine has become a critical strategy to improve access to diabetes care while simultaneously supporting necessary physical distancing during the pandemic, representing one of the biggest and swiftest care transformations worldwide. There is a large body of evidence for the efficacy of traditional telemedicine in diabetes care (real-time audiovisual visits), showing parity in outcomes with traditional office visits (3). The advent of connected technologies such as insulin pumps, smart insulin pens, and continuous glucose monitors lend themselves to virtual modalities of sharing patient information. For patient populations with less technology literacy or lower wi-fi bandwidth, telephone visits are also possible to increase access to necessary care. Practices can choose from a number of technology platforms to facilitate telemedicine, including existing electronic health record (EHR) and other commercially available tools. Options for sharing glucose data include newer cloud-based platforms versus traditional self-reporting of blood sugar logs; however, technological approaches must match patient population capabilities. In addition to medical visits, newer models of virtual care delivery by the entire multidisciplinary team has gained favor. Tele-education, nutrition, and psychology are among the newest telemedicine models that may bridge longstanding gaps in required multidisciplinary support and collaboration in diabetes care. Continuation of health technology platform support and collaboration will be needed to promote infrastructure building and sustainability beyond the pandemic.

### eConsultation

eConsults are another innovation that greatly increases the collaboration of different service lines, providing access to high-quality endocrinology care, and was particularly useful during the pandemic. eConsults are patient-specific, asynchronous virtual communication between a specialist and referring provider (typically primary care) that occurs within a shared EHR or other secure electronic platform. The benefits of eConsults across specialties are well documented and include increased access to specialty care (4), provider satisfaction (5), and educational benefits for primary care providers (6). More specifically, eConsults for diabetes care in the Veterans Administration Health System have resulted in more rapid access to specialty care with comparable clinical outcomes to in-person care (7). In COVID-19, both outpatient and inpatient eConsults have been pivotal in enabling the specialty endocrinologist to provide timely and efficient consultation. In addition, it enables endocrinologist-led foundational education to providers who benefit from real-time feedback on cases, which can bring back joy to endocrinology work.

### Project ECHO

Project ECHO (8) is a scalable, global, evidence-based telementoring program that aims to build internal capacity of primary care providers for diabetes care through ongoing case-based learning. The format includes a “hub” (eg, endocrinologist) that engages with a geographically dispersed group of primary care clinicians (spokes) by video, who present their cases on a routine basis and progressively build local diabetes expertise. Initial centralization of knowledge acquisition followed by dissemination to multiple primary care providers enhances the reach of endocrinology far beyond specialist capacity, holding great promise for continued reach of new therapeutics to the masses. Diabetes/endocrinology ECHO programs are currently active in 4 countries and are of greatest value when targeting primary care providers with limited access to specialty care (e.g., community health centers) (9).

### Team-based care

Within a team-based care model, a variety of team members have specific roles and responsibilities for ensuring patients receive optimal diabetes care. A shift from “the doctor takes care of patients and delegates significant work to team members” to “*we* take care of patients” (10) promotes collaborative care that can reduce burnout from clinical care demands while enhancing communication and access to quality diabetes care. During COVID-19, with virtual care models, the dissolution of in-person huddles and team meetings has made it difficult to sustain team-based care approaches; however, these shortcomings have been balanced with more access to ancillary services than previously. More innovation to enhance virtual communication among care teams is needed to provide optimal multidisciplinary diabetes care.

### Pharmacist-led care

The physician-pharmacist collaborative model in primary care has been shown to be efficacious in many chronic diseases, including diabetes. Clinical pharmacists have a PharmD degree and generally complete 1 to 2 years of postgraduate residency training. The pharmacist is embedded in the primary care or endocrinology practice and provides expertise in medication management for patients with complex diabetes, including initiation and titration of pharmacotherapy. Pharmacists are able to offload busy providers by managing a dedicated panel of patients and identifying and addressing barriers to medication adherence, while providing high-quality specialty diabetes care that may not be available elsewhere (11). A retrospective study of a pharmacist-physician collaborative care model in an integrated health system showed significant improvements in HbA1c, blood pressure, and cholesterol control as well as a 23% reduction in hospitalizations in the collaborative care patient group compared with usual care (12). Additionally, pharmacist collaborative models have been shown to improve outcomes for patients with type 1 diabetes (13).

### Pediatric to adult care transition

The transition from pediatric to adult care poses unique challenges to emerging adults with type 1 and type 2 diabetes who may struggle with the transition to independence in diabetes self-care. Care coordination between pediatric and adult systems is the foundation upon which successful transition can occur. However, communication and coordination are often missing because of EHR incompatibility for information transfer and deep cultural divides in pediatric and adult care paradigms that can be jarring for patients to shift between. Warm handoffs and transition navigator programs have demonstrated improved collaboration and access to adult care (14, 15). One-page health care transition summary documents are low-cost, efficient transfers of information that bridge providers and enable better therapeutic relationships with patients. Multidisciplinary team-based support including dieticians, educators, and psychologists can offer much needed care coordination and leverage virtual resources, which are now more readily available in COVID times. Additionally, virtual conferencing among patient, pediatric, and adult provider as a means of orientation that was not feasible before. As rates of youth-onset type 2 diabetes and young adult-onset type 1 diabetes increase, transition care models will need to be designed for sustainability and implementation in lower resourced settings.

## Threats to Innovation and Sustainability

Although innovative models of diabetes care were available in pre-COVID times, dissemination was limited because of a host of issues, some of which may reemerge once the pandemic ends. Barriers include: (1) lack of generalizable payment models; (2) slow technology integration into health care; (3) real-world implementation challenges; (4) a dearth of rigorous outcomes and cost data; (5) workforce shortages from burnout; and (6) no infrastructure for sustainability of innovative models.

Although some countries already had payment models for innovative diabetes care and more have adopted compensation strategies during the pandemic, it remains unclear whether coverage of innovative services will continue. Efforts to innovate care have typically been limited to institutions or practitioners with external funding or to integrated payor-provider systems, leading to a lack of generalizability and sustainability. Of late, health systems and stakeholders have increased their appetite for care strategies that work to improve medical outcomes and cut costs, even if not traditionally implemented. However, sustainability of pay models will rely on rigorous and large-scale outcome and cost data, which so far remains scarce.

In addition, lack of a roadmap for sustainability of innovation continues to threaten real change. Features of EHR workflows that facilitate telemedicine and e-Consults require extensive builds or funds to partner with third-party companies. Hardware and software support in addition to training on new technologies requires financial and staff resources that most nonacademic institutions are not able to provide. Lack of interoperability of different EHRs limits communication between care sites that are not part of the same institution. Continually siloed health centers and cultural differences in paradigms of care prevent different provider groups (for example, pediatric and adult counterparts or primary and endocrine care) from working together, even within the same health system. These systems of care, if not altered in more permanent ways, will continue to challenge foundational changes.

Moreover, continuing to align with organizational priorities will allow for continual support of strides made in health care. However, making a business case for these innovations is difficult. Many innovations require up-front investment and continued support from health care or governmental organizations. Endocrinologists also often lack the training, vocabulary, and pilot data to effectively advocate for budgets for innovative ideas. Moreover, the success of business cases somewhat relies on the environment in which the care is being practiced, whether fee for service or value-based system. This will continue to be relevant after the pandemic ends.

Finally, maintaining an impassioned and highly skilled workforce of endocrinologists to carry innovation forward remains the ultimate challenge. Before the pandemic and even more so now because of cost-limiting measures, both endocrinology teams and operations staff can be overwhelmed with routine work, having little time or resources to pursue innovation, even if the interest is there. Overall, fewer trainees are entering the field of endocrinology, limiting the influx of new ideas that are necessary for innovation. Although diabetes care can attract trainees from multiple disciplines, the increased administrative aspects of outpatient care greatly diminish the joys of work, leading to increased rates of burnout and mental health issues.

## Recommendations for a Better New Normal

To promote lasting health care transformation and create a better new normal for the health care community and our patients with diabetes, we must create systems that promote pathways for success and sustainability. Following are recommendations for all levels of stakeholders, which will help to ensure that innovation is sustained beyond the current pandemic. Professional Societies:

- Create virtual platforms, networks, and in-person meeting opportunities for health care practitioners to share practice transformation ideas and pilot project results
- Enhance training opportunities in quality improvement methodologies and formulation of the business case for innovation
- Leverage professional society relationships to advocate at the governmental level for alternative reimbursement models that facilitate innovation
- Encourage collaboration of primary care and specialty care professional societies to foster cross-collaborative networks such as quality improvement learning collaboratives, joint national meetings, and shared practice guidelines
- Foster technology-healthcare industry collaboration, and integration of health care redesign approaches into clinical practice settings

Healthcare Institutions and Practices:

- Encourage health care practitioners to incorporate quality improvement and implementation science methods in routine care
- Allocate philanthropic funding to promote clinical innovation and obtain pilot outcomes and cost data that can garner longstanding institutional or payor support
- Provide time for regular practice meetings to discuss innovative solutions to common clinical challenges supported through data collection
- Foster mentorship of clinicians to identify clinical improvements
- Enable multidisciplinary collaboration with service lines outside of endocrinology to build and sustain infrastructure for communication and innovation
- Encourage beta testing and early stage collaboration with health technology companies to introduce nontraditional frameworks of care

Health Care Practitioners:

- Seek virtual training opportunities in quality improvement methodology and dissemination-implementation science
- Search for mentorship and collaboration within or outside practice setting to discover best practices and learnings from failed experiences
- Understand organizational priorities and align initiatives by actively approaching administrators
- Recruit multiple team members (eg, nursing, practice managers, allied health care practitioners) to create a unified voice in advocating for change

## Conclusions

Faced with an unprecedented public health threat with the arrival of the COVID-19 pandemic, the value of innovative models of diabetes care is clearer than ever before. The pandemic has presented a disruptive force to health care worldwide. The response has been an embrace of innovation in diabetes care. Longstanding challenges were overcome seemingly overnight, showing us the great potential of innovative models. These successes in implementation have yet to be fully scaled, although with each passing month, we get closer to a better new normal. We are on the brink of a complete redesign in health care. Advocacy and community will be tantamount to the continued forward momentum we have gained. We must monopolize on this unique opportunity to reshape our current history into a positive and hopeful future.

## Data Availability

Data sharing is not applicable to this article as no datasets were generated or analyzed during the current study.

## Abbreviations

COVID-19: coronavirus disease 2019
EHR: electronic health record

## Additional Information

***Disclosure Summary:*** R.G. is on the advisory board of Onduo, Lark, Vida Health, Form Health, and Health Reveal. No other authors have related conflicts.
